# ToxRefDB v2.1: update to curated *in vivo* study data in the Toxicity Reference Database

**DOI:** 10.3389/ftox.2023.1260305

**Published:** 2023-09-11

**Authors:** Madison Feshuk, Lori Kolaczkowski, Sean Watford, Katie Paul Friedman

**Affiliations:** ^1^ Center for Computational Toxicology and Exposure, Office of Research and Development, U.S. Environmental Protection Agency, Research Triangle Park, Washington, DC, United States; ^2^ National Student Services Contractor, Oak Ridge Associated Universities, Oak Ridge, TN, United States; ^3^ Center for Public Health and Environmental Assessment, Office of Research and Development, U.S. Environmental Protection Agency, Washington, DC, United States

**Keywords:** database, toxicity, *in vivo*, reference, NAMs, curation

## Abstract

The Toxicity Reference Database (ToxRefDB) contains *in vivo* study data from over 5,900 guideline or guideline-like studies for over 1,100 chemicals. The database includes information regarding study design, chemical treatment, dosing, treatment group parameters, treatment-related (significantly different from control) and critical (adverse) effects, guided by a controlled effect vocabulary, as well as endpoint testing status according to health effects guideline requirements. ToxRefDB v2.1 is an update to address a compilation error found in ToxRefDB v2.0 that resulted in some effects being inadvertently omitted from the database. Though effect data has been recovered, no new studies were added. The recovered data improves the utility of ToxRefDB as a resource for curated legacy *in vivo* information, which enhances scientific confidence *in vitro* high-throughput screening of chemicals and supports retrospective and predictive toxicology applications for which outcomes in traditional regulatory toxicology studies serve as reference information.

## Introduction

One component of building scientific confidence in new approach methodologies (NAMs) for toxicology is comparison of results to *in vivo* studies. Further, scientific confidence in NAMs can be benchmarked against an evaluation of *in vivo* study performance for various types of toxicity testing (Pham et al., 2020; USEPA, 2021). However, such efforts require NAM and animal study data to be computationally accessible and interoperable. The Toxicity Reference Database (ToxRefDB) aggregates *in vivo* data from over 5,900 studies for over 1,000 chemicals (Martin et al., 2009a; Martin et al., 2009b; Watford et al., 2019).

ToxRefDB v2.1 provides recovered data and improves the utility of ToxRefDB as a resource for curated legacy *in vivo* toxicity information. The error resolved in ToxRefDB v2.1 is prevented in future database releases via changes in the curation workflow; moving forward, an application-driven workflow with the Data Collection Tool (DCT) will be utilized for loading curated information to ToxRefDB and will support a sustainable release cycle. The DCT is an Oracle APEX software developed for toxicity study extraction from legacy documents with enhanced quality control and data provenance capabilities. Continued expansion of chemical and study data in ToxRefDB increases its utility as a resource for retrospective analyses of guideline toxicology information that lay the foundation for acceptance of NAMs as well as development of new predictive tools.

This data report will provide an overview of ToxRefDB and describe the significance of the v2.1 data update.

## Methods

**FIGURE 1:**
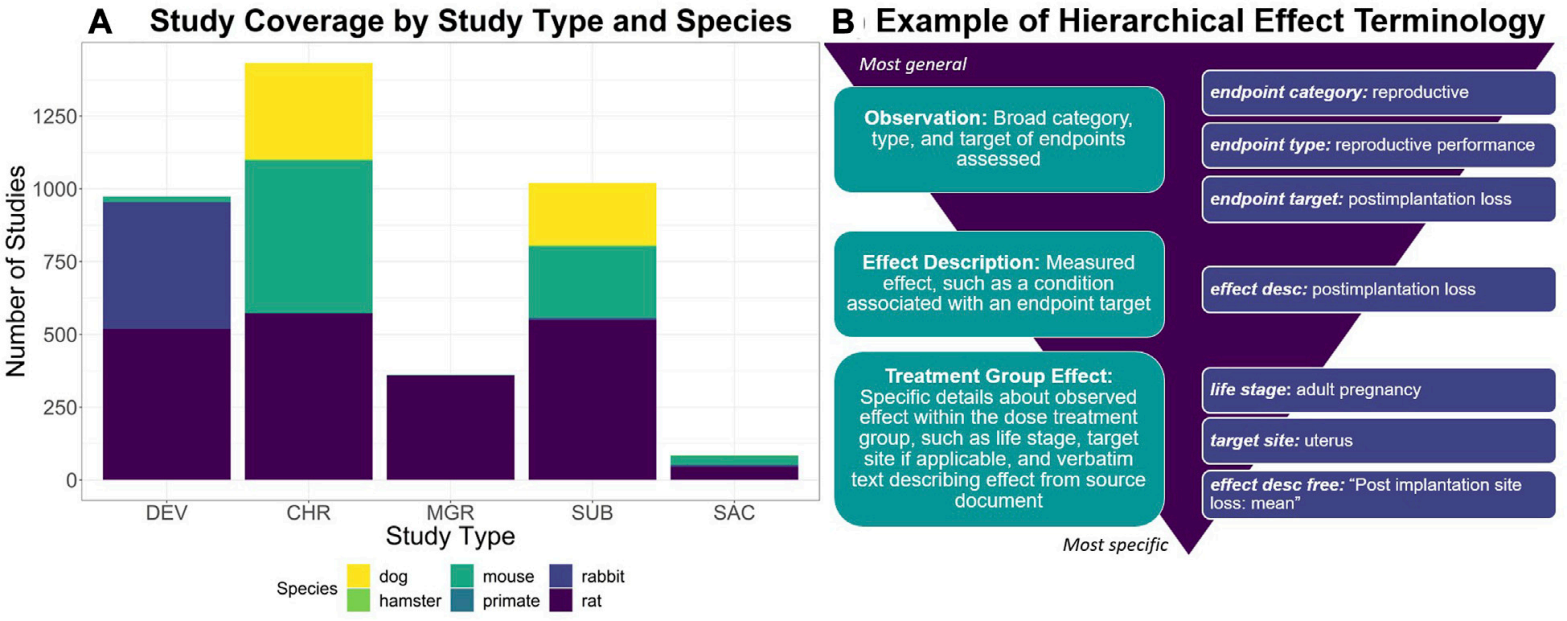
**(A)** To describe the ToxRefDB data landscape, this stacked bar chart displays the number of studies by study type and species. Study type abbreviations are as follows: Chronic (CHR), Prenatal-Developmental (DEV), Multigeneration Reproductive (MGR), Subacute (SAC), and Subchronic (SUB). **(B)** To demonstrate ToxRefDB’s hierarchical effect terminology, one effect example “postimplantation loss”, as commonly measured in reproductive exposure studies such as MGR and DEV, is described. The finding will be recorded as is in the “effect description free” field, which is the verbatim wording used in the study report. The remaining fields are part of the ToxRefDB controlled terminology. The endpoint category is reproductive, the endpoint type is reproductive performance, the endpoint target is postimplantation loss, the effect description is postimplantation loss, and the specific observation of “postimplantation loss” was made in the adult pregnancy life-stage at the specific target site, the uterus.

Developed via a manual curation workflow, ToxRefDB serves as a resource for study design, quantitative dose response, specific effects, and endpoint testing status information given guideline specifications from the US EPA’s Series 870 Health Effects Test Guidelines or the National Toxicology Program (NTP). These study types are used commonly in regulatory toxicology applications to assess chemical safety. Study coverage, as shown in Figure 1A, includes a variety of repeat dose study designs, such as chronic, subchronic, subacute, prenatal developmental, and multigeneration reproductive, conducted in various species and utilizing various administration routes, predominately oral. Many of the studies (over 3,000) come from the US EPA’s Office of Pesticide Programs (OPP) review of registrant-submitted toxicity studies, known as data evaluation records (DERs). Indeed, 90% of studies with completed curation correspond to pesticide actives and inerts. Continued curation efforts have expanded ToxRefDB to include toxicity studies from additional sources, including NTP reports, pharmaceutical pre-clinical studies, and guideline-like open literature. An important feature of ToxRefDB is its use of controlled vocabularies for enhanced data quality and interoperability. The custom ToxRefDB vocabulary was developed to match guideline specifications and has been mapped to appropriate terms in the United Medical Language System (UMLS) vocabulary. An example of the hierarchical effect terminology is provided in Figure 1B for post-implantation loss during reproductive exposure.

ToxRefDB v2.1 is a data update that captures curated effects that were not fully reflected in ToxRefDB v2.0. In ToxRefDB v2.0, compilation script issues resulted in some extracted values failing to import properly into the MySQL database from the originating AccessDB curation files. This update recovers effect data for 594 studies (predominantly multi-generation reproductive (MGR) and developmental (DEV) studies), although no additional study curation was performed. The v2.1 update includes recovered data from previous curations for an additional 5,226 dose treatment groups with ultimately 21,756 recovered effects added across the database. As ToxRefDB v2.0 was missing some effect data, the v2.1 update increases the fidelity of the database by providing a more complete curation of legacy animal toxicity studies conducted. This update was necessary to serve as the foundation for future versions using a new curation workflow.

## Version comparison analysis

Given the extent of the recovered data, study and chemical-level analyses were conducted to evaluate the potential impact on the database using aggregated points of departure (PODs) as a metric for comparison. The following POD types were derived based on the extracted effect data at both the chemical and study-level: the Lowest Effect Level (LEL: the lowest dose with observed treatment-related effects), the Lowest Observed Adverse Effect Level (LOAEL: the lowest dose with observed critical effect), the No Effect Level (NEL: the highest dose with no observed effects, as inferred from LEL), and the No Observed Adverse Effect Level (NOAEL: the highest dose with no observed critical effects, as inferred from LOAEL), where “treatment-related” indicates the effects were statistically significant from the control and “critical” indicates that effects were considered adverse by toxicologist reviews of the study findings.

**FIGURE 2:**
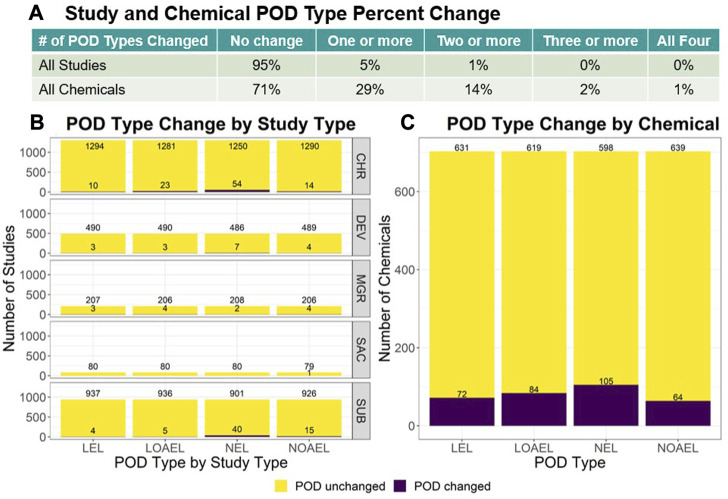
For each study and chemical in v2.0 and v2.1, the lowest LEL, lowest LOAEL, highest NEL, and highest NOAEL were identified for comparison. **(A)** The table shows the number of changed values across POD types. Options include: “No change”, or change in “One or more”, “Two or more”, “Three or more”, or “All four” POD types. Only 5% of all studies had a change in 1 or more PODs. **(B)** When data is stratified by study type, the study type with the most change were CHR and SUB study types with SAC showing the least amount of change. **(C)** 29% of chemicals across all study types had a change in 1 or more POD types, with only 2% showing change in 3 or more. These study-level comparisons do not consider the PODs added for the 594 studies with effects. Chemical-level change drastically decreases when subset to exclude the PODs added for the 377 DEV and 123 MGR studies that were most impacted by compilation error and have the most recovered data.

For each study and chemical across v2.0 and v2.1, the lowest LEL, lowest LOAEL, highest NEL, and highest NOAEL were compared. The summary results are reflected in Figure 2 whereas the full analysis is described within the ToxRefDB v2.1 release note. Study-level change showed only 5% of all studies had a change in 1 or more POD types, with most change observed in chronic (CHR) and subchronic (SUB) studies and within the NEL POD type. Chemical-level change found 29% of chemicals *across all study types* had a change in 1 or more POD types, with only 2% showing a change in 3 or more POD types. In both the study and chemical-level analyses, the mean magnitude of change values fall under 1 log_10_-mg/kg/day. The overall study-level median magnitude of change was 0.34 ± 0.36 log_10_-mg/kg/day (median ± median absolute deviation, or MAD), whereas the overall chemical-level median magnitude of change was 0.30 ± 0.26 log_10_-mg/kg/day (median ± MAD).

Given the high frequency of changed POD types and presence of magnitude of change values observed in the chemical-level analyses, the data was then subset to consider repeat dose studies, such as CHR, SUB, and sub-acute (SAC) study types, and the new PODs added from the 594 predominantly DEV and MGR studies were excluded. This subsequent analysis showed that 15% of chemicals had a change in 1 or more POD types with only 5% showing a change in 2 or more, which is indicative that the initial high chemical level change observed could be attributed to the added MGR and DEV data. These reproductive study types often include different dose selection as well as potentially sensitive lifestages, which may shift results by chemical when summarizing all quantitative treatment-related data available for that chemical. The recovered data in ToxRefDB v2.1 resulted in small overall potency shifts as reflected by the study and chemical level POD changes. ToxRefDB v2.1 represents an iterative improvement to a high-quality resource for curated legacy *in vivo* information needed for retrospective and predictive toxicology applications.

## Availability and implementation

Visit this webpage (https://www.epa.gov/chemical-research/downloadable-computational-toxicology-data) to download the ToxRefDB v2.1 MySQL database package, including release note describing the analysis conducted and an accompanying ToxRefDB user guide, containing installation and sample query instructions. ToxRefDB derived point-of-departure values at the chemical and study level are included in the summary-level database, the Toxicity Value Database (ToxValDB), which aggregates hazard data from multiple public sources and is surfaced on the CompTox Chemicals Dashboard (CCD) on the Hazard tab (full ToxRefDB information will be available for download from Batch Search in a future CCD release). All chemicals associated with curations in ToxRefDB v2.0-1 are provided as a chemical list on the CCD (https://comptox.epa.gov/dashboard/) under the TOXREFDB2 chemical list.
